# Comparison of the relative diagnostic performance of ^68^Ga-DOTA-IBA and ^18^F-NaF for the detection of bone metastasis

**DOI:** 10.3389/fonc.2024.1364311

**Published:** 2024-03-22

**Authors:** Jia Deng, Jian Yang, Yingwei Wang, Guangfu Liu, Yue Chen

**Affiliations:** ^1^ Department of Nuclear Medicine, The Affiliated Hospital of Southwest Medical University, Luzhou, Sichuan, China; ^2^ Nuclear Medicine and Molecular Imaging Key Laboratory of Sichuan Province, Luzhou, Sichuan, China; ^3^ Nuclear Medicine Institute of Southwest Medical University, Luzhou, Sichuan, China

**Keywords:** DOTA-IBA, ^68^Ga, PET/CT, ^18^F-NaF, bone metastases

## Abstract

**Purpose:**

We aimed to compare the relative diagnostic efficacy of ^68^Ga-Labeled DOTA-ibandronic acid (^68^Ga-DOTA-IBA) to that of^18^F-NaF PET/CT as a mean of detecting bone metastases in patients with a range of cancer types

**Methods:**

This study retrospectively enrolled patients with bone metastases associated with various underlying malignancies. All patients underwent both ^68^Ga-DOTA-IBA and ^18^F-NaF PET/CT scans. Histopathology and follow-up CT or MRI imaging results were used as reference criteria, with a minimum follow-up period of 3 months. The maximum Standardized Uptake Value (SUVmax) and number of bone metastases were recorded. The Target-Background Ratio (TBR) was calculated along with the detection rate, sensitivity, specificity, positive predictive value (PPV), negative predictive value (NPV), and accuracy of ^68^Ga-DOTA-IBA and ^18^F-NaF PET/CT imaging for overall and partial primary solid tumor bone metastases. Pearson chi-square test, McNemar test, and Kappa test was conducted to assess the correlation and consistency of diagnostic efficiency between the two imaging agents. Receiver Operating Characteristic curve (ROC curve) was performed to compare diagnostic performance and the area under the curve of the two imaging agents, determining optimal critical values for SUVmax and TBR in diagnosing bone metastasis. Differences in SUVmax and TBR values between the two imaging agents for detecting bone metastases were analyzed using the Wilcoxon signed rank test. The difference was statistically significant when P < 0.05

**Results:**

A total of 24 patients (13 women and 11 men) were included in this study, with a mean age of 52 (interquartile range, 49-64 years). The detection rate, sensitivity, specificity, PPV, NPV, accuracy, and AUC of ^68^Ga-DOTA-IBA and ^18^F-NaF PET/CT for bone metastases were 81%, 90%, 62%, 95%, 43%, 88%, 0.763, and 89%, 99%, 59%, 95%, 89%, 95%, 0.789, respectively. There was no significant difference between the two imaging methods (P < 0.01), and there was a significant correlation (X^2^=168.43, P < 0.001) and a strong consistency (Kappa=0.774,P < 0.001) between the diagnostic results of the two imaging agents. The SUVmax values of lesions measured by ^68^Ga-DOTA-IBA and ^18^F-NaF imaging in 22 patients with bone metastasis were 5.1 ± 5.4 and 19.6 ± 15.1, respectively, with statistically significant differences (P<0.05). The TBR values of the two imaging methods were 5.0 ± 5.0 and 6.7 ± 6.4, respectively, with statistically significant differences (P<0.05). The AUC of the SUVmax of ^68^Ga-DOTA-IBA and ^18^F-NaF curves were 0.824 and 0.862, respectively, with no statistically significant difference (P=0.490). No significant difference was found in the AUC of the TBR of ^68^Ga-DOTA-IBA and ^18^F-NaF (0.832 *vs* 0.890; P=0.248). Subgroup analysis showed significant correlation between the two imaging agents in the diagnosis of bone metastases in lung cancer and breast cancer, with consistent diagnostic results. However, in the diagnosis of bone metastases in prostate cancer, there was a significant difference (P<0.001) and lack of consistency (P=0.109)

**Conclusion:**

The diagnostic efficacy of ^68^Ga-DOTA-IBA for bone metastasis lesions is comparable to that of ^18^F-NaF. This finding holds significant clinical importance in terms of diagnosis of bone metastasis and selecting treatment plans for patients with malignant tumors

## Introduction

Bone is the third common metastatic site of various solid tumors (including lung, breast, prostate, colon, thyroid, gynecology and melanoma), and notably arising from the breast (70%), prostate (85%) and lung (40%) (1). Unfortunately, once cancer spreads to the bones, it is difficult to cure and is associated with a range of complications, including pain, increased risk of fractures, and hypercalcemia, all of which significantly diminish the quality of life for affected patients (2, 3).

Molecular imaging using bone targeted tracers has been applied in clinical practice for nearly 50 years and still plays an important role in the diagnosis and follow-up of bone metastases. It includes ^99m^Tc-bisphosphonate for bone scanning and ^18^F-NaF and ^18^F-FDG for PET/CT (4–6). However, ^18^F-NaF requires a medical cyclotron for production, which limits its clinical application. Nevertheless, it generally exhibits higher sensitivity and specificity compared to ^99m^Tc-MDP bone scanning and ^18^F-FDG (7–9). However, bisphosphonates have great value in the diagnosis and treatment of skeletal diseases, as they have a strong affinity for bones (10). Ibandronic acid (IBA) is a third-generation amino-bisphosphonate with antiresorptive and antihypercalcemic properties. We successfully synthesized chelate DOTA-containing ligand DOTA-IBA. Basic experimental articles on ^68^Ga-DOTA-IBA and ^177^Lu-DOTA-IBA have been published and proved to provide a set of potential theranostic radiopharmaceuticals and may have a good prospect for the management of bone metastasis (11–13). Gallium-68 (^68^Ga) is commonly used as a positron imaging agent in clinical practice and can be obtained using a germanium gallium generator (^68^Ge/^68^Ga). Furthermore, when assessing subsequent radionuclide therapy the imaging results of the ^68^Ga tracer outperform those of the ^18^F tracer (14). Similarly, The utilization of ^68^Ga/^177^Lu-DOTA-IBA can be beneficial in the diagnosis and assessment of bone metastases in malignant tumors. ^68^Ga-DOTA-IBA not only enables positron imaging but also capitalizes on IBA’s high adsorption rate on bones. As a novel bone-seeking positron radioactive drug, further research is needed to evaluate the diagnostic performance of ^68^Ga-DOTA-IBA. As such, we herein performed a comparative analysis of the relative performance of ^68^Ga-DOTA-IBA and ^18^F-NaF PET/CT for the detection of bone metastases in patients with a range of tumor types.

## Patients and methods

### Patients

This retrospective study was approved by the Ethics Committee of the Affiliated Hospital of Southwest Medical University (Ethics committee approval No.: KY2022114; Clinical trial registration No.: ChiCTR2200064487) and was conducted between September 2021 and May 2023. Patients with malignant tumors who underwent ^68^Ga-DOTA-IBA and ^18^F-NaF PET/CT imaging were enrolled in this study. The interval between the two examinations was less than 7 days. All of the above patients were recruited under written consent. The exclusion criteria were the skeletal metastatic lesions had received treatment before imaging analyses and patients who were lost to follow-up.

### PET/CT imaging acquisition

The patients were weighed and no other special patient preparation was needed before the examination. ^18^F⁃NaF was produced by Siemens cyclotron. The ^68^Ga-DOTA-IBA labeling was carried out according to the method described previously (9). The radiochemical purity of ^18^F⁃NaF and ^68^Ga-DOTA-IBA were both exceeded 95%. Intravenous doses of ^18^F-NaF and ^68^Ga-DOTA-IBA were 3.7 MBq/kg(0.1mCi/kg) and 1.85MBq/kg(0.05mCi/kg), respectively. PET/CT scans (uMI780, United Imaging Healthcare) were performed approximately 45-60 minutes after intravenous administration. The CT scan was performed and acquired according to the following parameters: tube voltage, 120 kV; current, 120 mA; layer thickness 3.00mm, layer spacing 5 mm, pitch 0.813. The PET scan was then performed in 3D acquisition mode on the same bed as the CT. When the reconstruction was complete, the images were processed using PET/CT post-processing software.

### Imaging interpretation

^68^Ga-DOTA-IBA and ^18^F-NaF PET/CT images were independently interpreted in a visual and semi-quantitative manner by two experienced nuclear medicine physicians. Based on the results of the ^68^Ga-DOTA-IBA and ^18^F-NaF imaging agents, the lesions were categorized into bone metastases, benign bone lesions, and normal bones. A lesion was defined as a region of abnormal radioactivity when the uptake value was significant higher than the surrounding normal bone background.

### Reference standard

Bone metastases were confirmed by pathology. However, due to technical and ethical issues, pathological findings could not be performed on all suspected involved lesions. We used the results of imaging examination results (BS, CT, MRI, or PET/CT) and clinical follow-up (physical signs and follow-up imaging examination) as the reference standards. The follow-up time was at least 3 months. When pathological results are lacking, bone metastases were judged on the findings of typical performance (extensive bone metastases throughout the skeleton) on PET/CT and corresponding characteristic morphologic findings of metastasis on the CT component as well as the improvement or progression of bone metastatic lesions following treatment at follow-up imaging examination results. The lesions were categorized as benign lesions owing to typical appearance on imaging examination and no progressive performance was found during the follow-up period.

### Statistical analysis

Statistical analysis was performed using SPSS 27.0 (IBM, Armonk, NY, USA). All quantitative information was expressed as the mean ± standard deviation. We compared the diagnostic performance and diagnostic efficacy of the two imaging modalities using Pearson chi-square test, McNemar test and the receiver operating characteristic curve (ROC curve). The Kappa test was utilized to assess the consistency of the diagnostic performance between the two imaging modes. A kappa value of ≥ 0.75 indicates good consistency, while a value between 0.4 and 0.75 suggests average consistency. A kappa value less than 0.4 indicates poor consistency. Comparison of SUVmax and TBR for ^18^F-NaF and ^68^Ga-DOTA-IBA for bone metastatic was performed using the Wilcoxon test. The ROC curve was used to obtain the optimal critical values for SUVmax and TBR diagnosis of bone metastases, and the differences in area under curve (AUC) were compared. P < 0.05 was considered to indicate a statistically significant difference.

## Results

### Patient characteristics

In total, 24 patients (13 women and 11 men) were included in this study, with a mean age of 52 (interquartile range, 49-64 years). There were 12 (50%) patients with lung cancer, 5 (21%) with breast cancer, 2 (8%) with prostate cancer, 2 (8%) with renal cancer, 1 (4%) with stomach cancer, 1 (4%) with pancreatic cancer, and 1 (4%) with rectal cancer. Patient characteristics are summarized in **Table 1**.

**Table 1:** Summary of Patient Characteristic.

Characteristic	Value
**No.Patient**	24
Age,y	
Median	52
Interquartile rage	49-64
Sex	
Male	11
Female	13
Diagnosis	
Lung cancer	12
Breast cancer	5
Prostate cancer	2
Renal cancer	2
Stomach cancer	1
Pancreatic cancer	1
Rectal cancer	1
Scanning purposes	
Staging at initial diagnosis	2
Restaging after therapy	22

### Comparison of ^68^Ga-DOTA-IBA versus ^18^F-NaF PET for detecting bone metastases

**Figure 1:**
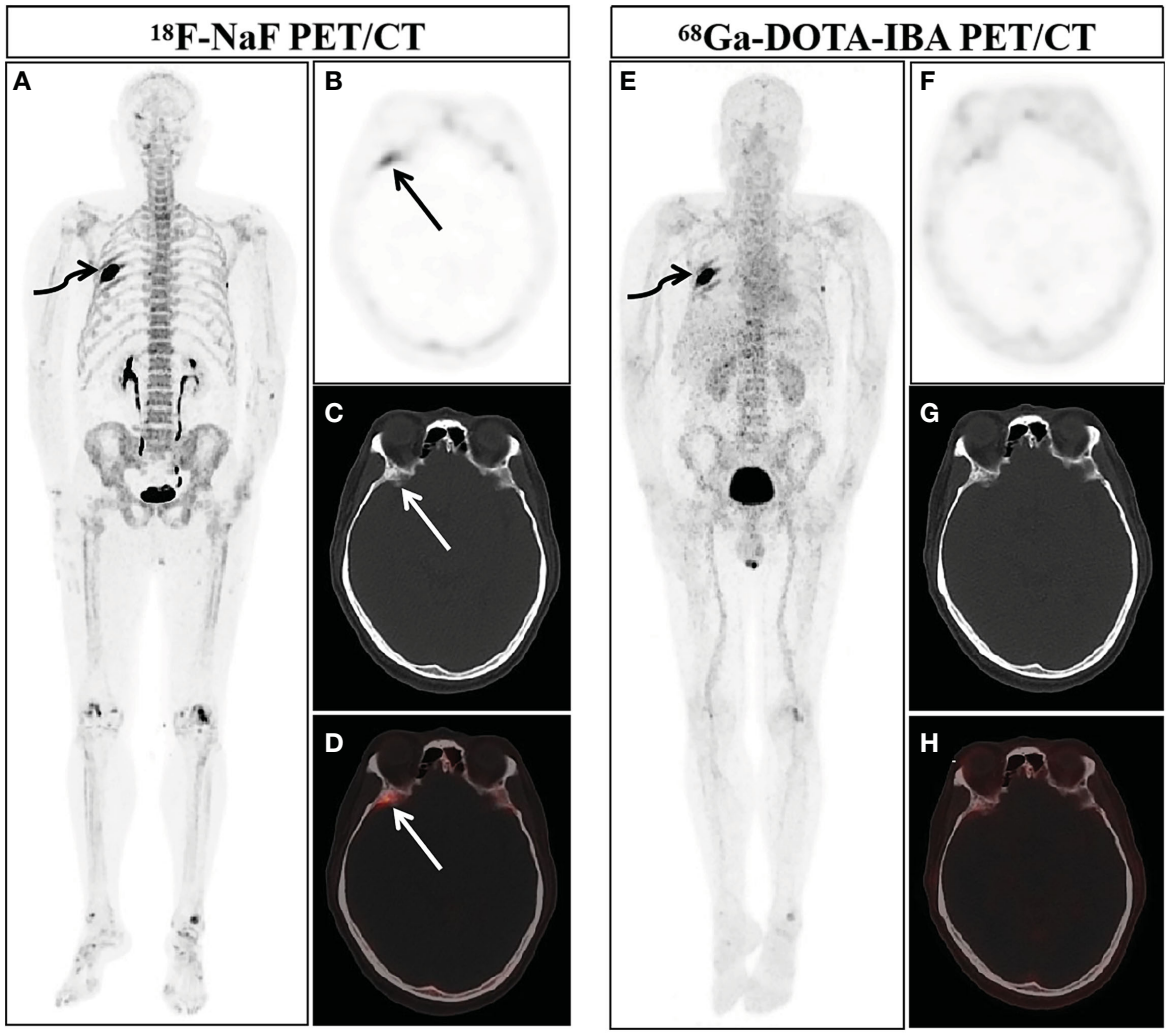
A 58-year-old man newly diagnosed with lung cancer was included in our study and underwent ^18^F-NaF and ^68^Ga-DOTA-IBA PET/CT. The MIP images of ^18^F-NaF **(A)** and ^68^Ga-DOTA-IBA **(E)** PET/CT both showed an increase in imaging agent uptake in the right rib (curved arrows). Combined with his history of malignant tumor and typical osteolytic bone destruction with soft tissue shadows, the diagnosis of bone metastasis was considered. The MIP image **(A)** and axial views (**B**: PET image; **C**: CT scan; **D**: PET/CT fused image) of ^18^F-NaF showed the increased imaging agent uptake of the right sphenoid wing (straight arrow, SUVmax 5.3), but no significant bone abnormalities were observed. ^68^Ga-DOTA-IBA PET/CT MIP **(E)** and axial views (**F**: PET image; **G**: CT scan; **H**: PET/CT fused image) revealed there was no significant abnormal increase in imaging agent uptake on the right sphenoid wing. After nearly three months of follow-up, according to the patient’s CT examination, there were no obvious abnormalities in the bone, and we believed that this was physiological uptake.

In our study, due to ethical and practical reasons (for example, patients with a history of pathologically diagnosed malignant tumors, multiple bone lesions, and they showed typical bone metastases in two or more imaging examinations), so there is no confirmation of bone metastasis based on biopsies. The final diagnosis of these metastases is based on comprehensive imaging findings (BS, CT, MRI or PET/CT) and clinical follow-up (physical signs and follow-up imaging examination). Bone metastases were both successfully detected by ^68^Ga-DOTA-IBA PET/CT and ^18^F-NaF PET/CT in 22 patients. A total of 252 lesions in 22 patients were diagnosed with bone metastasis. 29 lesions were categorized as benign lesions owing to typical appearance on imaging examination and no progressive performance was found during the follow-up period. 239 bone lesions were found to have increased uptake of ^68^Ga-DOTA-IBA. Among these ^68^Ga-DOTA-IBA-avid lesions, 228 bone lesions were considered to represent true positive lesions of bone metastasis, while 11 benign lesions with false positive uptake were identified. Benign lesions include degenerative osteophytes (2/11), arthritis (3/11), and fractures (6/11). In contrast, ^18^F-NaF detected 262 bone lesions with increased uptake, and 12 false positive lesions were found through ^18^F-NaF PET/CT imaging, including physiological uptake (1/12), arthritis (2/12), fractures (3/12), and degenerative osteophytes (6/12). **Figure 1** showed that the physiological uptake of right sphenoid wing of a patient was false positive in ^18^F-NaF PET/CT imaging, while the uptake of ^68^Ga-DOTA-IBA was not significantly increased.

**Figure 2:**
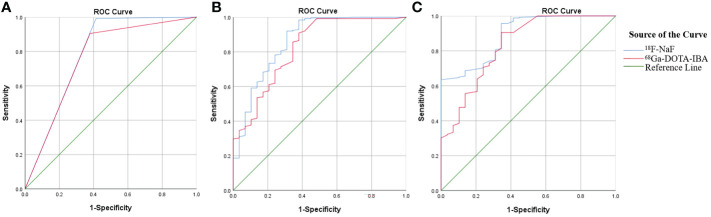
Comparison of ROC curves between ^68^Ga-DOTA-IBA and ^18^F-NaF PET/CT in diagnosing bone metastasis **(A)**; Comparison of ROC curves for diagnosing bone metastasis using SUVmax in ^68^Ga-DOTA-IBA and ^18^F-NaF PET/CT imaging **(B)**; Comparison of ROC curves for TBR diagnosis of bone metastasis in ^68^Ga-DOTA-IBA and ^18^F-NaF PET/CT imaging **(C)**.

The detection rate, sensitivity, specificity, positive predictive value (PPV), negative predictive value (NPV), accuracy, and the AUC of ^68^Ga-DOTA-IBA PET/CT and ^18^F-NaF PET/CT in the diagnosis of bone metastases were 81%, 90%, 62%, 95%, 43%, 88%, 0.763, and 89%, 99%, 59%, 95%, 89%, 95%, 0.789, respectively (**Table 2**). There was no statistically significant difference in diagnostic performance between the two imaging methods (P=1.00). Pearson chi-square test showed that there was a significant correlation between the diagnostic results of the two imaging agents (X^2^ = 168.43, P < 0.001). The diagnostic consistency of the two imaging methods for bone metastases was found to be good (Kappa=0.774, P<0.001). In addition, there was no statistically significant difference between the two imaging agents in diagnosing bone metastasis AUC (P=0.620) (**Figure 2A**).

**Table 2:** Diagnostic Efficacy of ^68^Ga-DOTA-IBA PET/CT and ^18^F-NaF PET/CT in Bone Metastases.

Index	Bone Metastasis	Final Result		Detection rate (%)	Sensitivity(%)	Specificity(%)	PPV (%)	NPV (%)	accuracy(%)	AUC	
		+	-								
^68^Ga-DOTA-IBA	+	228	11	81	90	62	95	43	88	0.763	P*=0.620
	–	24	18								
^18^F-NaF	+	250	12	89	99	59	95	89	95	0.789	
	–	2	17								

*P:Comparison between ^68^Ga-DOTA-IBA and ^18^F-NaF groups of AUC.

+:Diagnosed as bone metastasis.

-:Diagnosed as non bone metastasis.

### Comparative analysis of SUVmax of ^68^Ga-DOTA-IBA PET/CT and ^18^F-NaF PET/CT in bone metastasis

In our image analysis, physiological uptake areas in ^68^Ga-DOTA-IBA PET/CT is consistent with previous basic research (11). In general, ^68^Ga-DOTA-IBA is a radiopharmaceutical excreted through the kidney, with rapid soft tissue clearance and high bone uptake, and is retained for a long time in bone disease. The statistical analysis revealed that the SUVmax of ^18^F-NaF PET had a higher value for detecting bone metastasis compared to the SUVmax of ^68^Ga-DOTA-IBA PET (19.6 ± 15.1 *vs* 5.1 ± 5.4; P<0.05). To evaluate the diagnostic performance, ROC curve analysis was conducted using the SUVmax measurements from both ^68^Ga-DOTA-IBA and ^18^F-NaF imaging, along with the diagnostic results of all lesions (**Figure 2B**). The results indicated that the AUCs of SUVmax for diagnosing bone metastasis were 0.824 and 0.862 for ^68^Ga-DOTA-IBA and ^18^F-NaF, respectively. There was no statistically significant difference (P=0.490). Furthermore, the optimal diagnostic critical values were determined as 1.15 and 9.65 for ^68^Ga-DOTA-IBA and ^18^F-NaF, respectively (**Table 3**).

**Table 3:** Comparison Between ^68^Ga-DOTA-IBA and ^18^F-NaF using SUVmax in the Diagnosis of Bone Metastases.

Index	Cutoff	Sensitivity	Specificity	AUC	Youden index*	*P*	*P’* (Inter group comparison)
SUVmax-IBA	1.15	91%	62%	0.824	0.530	<0.001	0.490
SUVmax-NaF	9.65	92%	69%	0.862	0.611	<0.001	

*Youden index=Sensitivity+Specificity-1.

### Comparative analysis of TBR of ^68^Ga-DOTA-IBA PET/CT and ^18^F-NaF PET/CT PET/CT in bone metastasis

In the detection of bone metastasis, the TBR of ^18^F-NaF PET showed a slightly higher value compared to that of ^68^Ga-DOTA-IBA PET. This difference was found to be statistically significant (6.7 ± 6.4 *vs* 5.0 ± 5.0; P<0.05). ROC curve analysis was conducted to evaluate the diagnostic performance of TBR using both imaging agents, ^68^Ga-DOTA-IBA and ^18^F-NaF, for all lesions (**Figure 2C**). The AUC values for TBR in the diagnosis of bone metastases were 0.832 and 0.890, respectively, with corresponding optimal diagnostic cutoff values of 1.45 and 4.95 (**Table 4**). Notably, there was no significant difference in AUC between ^68^Ga-DOTA-IBA PET/CT and ^18^F-NaF PET/CT when using TBR for diagnosis (P=0.248).

**Table 4:** Comparison Between ^68^Ga-DOTA-IBA and ^18^F-NaF using TBR in the Diagnosis of Bone Metastases.

Index	Cutoff	Sensitivity	Specificity	AUC	Youden index*	*P*	*P’* (Inter group comparison)
TBR-IBA	1.45	91%	66%	0.832	0.560	<0.001	0.248
TBR-NaF	4.95	66%	100%	0.890	0.635	<0.001	

*Youden index=Sensitivity+Specificity-1.

### Analysis of the efficacy of ^68^Ga-DOTA-IBA PET/CT and ^18^F-NaF PET/CT imaging in the diagnosis of bone metastases from lung, breast and prostate cancer

According to the pathological types of primary tumors, we analyzed lung cancer (n = 12), breast cancer (n = 5) and prostate cancer (n = 2) with bone metastases.

Among 12 lung cancer patients, 11 developed bone metastases, with a total of 133 lesions diagnosed as bone metastases. Based on the lesions, 117 positive lesions were correctly detected by ^68^Ga-DOTA-IBA PET/CT, with 5 true negatives. ^18^F-NaF PET/CT detected 124 positive lesions, including 3 false positives. No significant difference in the diagnostic efficacy of the two imaging agents (P=1.00), while Pearson chi-square test and Kappa test showed a significant association and a moderate consistency in diagnosing bone metastases between the two imaging agents (X^2^ = 35.104, P<0.001; Kappa=0.493, P<0.001).

Among 21 lesions ultimately confirmed as bone metastases in 5 cases of breast cancer, both ^68^Ga-DOTA-IBA PET/CT and ^18^F-NaF PET/CT successfully detected 20 positive lesions. McNemar test revealed no significant difference in diagnostic efficacy between the two tracers (P=0.250). Pearson chi-square test and Kappa test indicated a significant association between the diagnostic results of the two tracers, with a strong consistency (X^2^ = 21.156, P<0.001; Kappa=0.767, P<0.001).

In 2 cases of prostate cancer patients, a total of 28 bone metastatic lesions were detected. ^18^F-NaF PET/CT correctly identified 28 positive lesions, while ^68^Ga-DOTA-IBA PET/CT successfully detected 13 positive lesions. Unlike lung cancer and breast cancer, McNemar test revealed a significant difference in the diagnostic efficacy of the two imaging agents (P<0.001). Pearson chi-square test and Kappa test indicated no significant association between the diagnostic results of the two imaging agents (X^2^ = 2.575, P=0.109), and there was no consistency in the diagnosis of bone metastasis between the two agents (Kappa=0.149, P=0.109). For detailed information on the detection rate, sensitivity, specificity, PPV, NPV, and accuracy of ^68^Ga-DOTA-IBA PET/CT and ^18^F-NaF PET/CT in diagnosing bone metastases in lung cancer, breast cancer, and prostate cancer, please refer to **Table 5**.

**Table 5:** Analysis of ^68^Ga-DOTA-IBA PET/CT and ^18^F-NaF PET/CT in the diagnosis of bone metastases of different solid tumors.

Tumor type	Tracers	Preliminary diagnostic results	Final diagnosis result		Detection rate (%)	Sensitivity(%)	Specificity(%)	PPV (%)	NPV (%)	Accuracy (%)	Pearson test (X^2^ value)	Kappa test (Kappa value)	McNemar test
			+	-									
Lung cancer	^68^Ga-DOTA-IBA	+	128	6	89	96	45	96	50	92	35.104 (p<0.001)	0.493 (p<0.001)	P=0.100
		–	5	5									
	^18^F-NaF	+	132	3	92	99	73	98	89	97			
		–	1	8									
Breast cancer	^68^Ga-DOTA-IBA	+	20	4	59	95	69	83	90	85	21.156 (p<0.001)	0.767 (P<0.001)	P=0.250
		–	1	9									
	^18^F-NaF	+	20	7	59	95	46	74	86	76			
		–	1	6									
Prostate cancer	^68^Ga-DOTA-IBA	+	13	1	41	46	75	93	17	50	2.575 (P=0.109)	0.149 (P=0.109)	P<0.001
		–	15	3									
	^18^F-NaF	+	28	1	88	100	75	97	100	97			
		–	0	3									

+:Diagnosed as bone metastasis.

-:Diagnosed as non bone metastasis.

## Discussion

Bone-targeting tracers are essential for diagnosing and monitoring bone metastases. The advancement of radioactive nuclides and molecular probes has resulted in their increased use in isotope therapy (15–19). This study aims to assess the effectiveness of ^68^Ga-DOTA-IBA PET/CT in detecting bone metastases and compare it with ^18^F-NaF PET/CT.

^18^F-NaF PET/CT is a well-established imaging tool for staging skeletal tumors, providing valuable information on disease location, extent, and treatment response. Its rapid extraction, minimal serum protein binding, and efficient clearance from soft tissues with high bone uptake result in superior image quality and consistency compared to traditional ^99m^Tc-MDP (8, 9, 20). Ibandronic acid (IBA), a third-generation amino diphosphate, possesses anti-resorption and anti-hypercalcemia properties. The ^68^Ga-DOTA-IBA used in this study is a new precursor that has been shown to have advantages over ^99m^Tc-MDP in detecting bone metastasis in previous studies (11, 13). Our preliminary research results indicated a significant association and strong consistency between ^68^Ga-DOTA-IBA and ^18^F-NaF PET/CT in diagnosing bone metastases. As a promising bone radiotracer, ^68^Ga-DOTA-IBA has the potential to offer diagnostic efficacy equivalent to that of ^18^F-NaF, suggesting it could become a routine diagnostic method or complementary tool for diagnosing bone metastases.

Furthermore, we made a subgroup analysis of lung cancer, breast cancer and prostate cancer with bone metastasis. However, because of the small sample size, we only made a preliminary analysis. The results revealed a significant correlation between the two imaging agents in diagnosing bone metastasis of lung cancer and breast cancer, with better consistency observed in breast cancer. However, there was no significant correlation or consistency in diagnosing bone metastasis of prostate cancer. Lung cancer, known for its osteophilic nature, can exhibit both osteolytic destruction and osteogenic metastasis. The specific pathological changes of bone metastasis of lung cancer can be affected by the degree of bone destruction, the scope and the speed of bone metastasis. Breast cancer, particularly invasive cases, commonly present with bone metastasis, predominantly showing osteolytic bone destruction accompanied by osteoblast reaction. Prostate cancer, typically slow-growing, may not be fatal in its primary form but can lead to severe complications and death when bone metastasis occurs, often displaying osteogenic changes (21). In our study, ^68^Ga-DOTA-IBA PET/CT were inconsistent with the higher sensitivity of ^18^F-NaF PET/CT in detecting bone metastases in prostate cancer. We believe this discrepancy may be due to the diffuse osteoblastic changes that often occur in the bones when prostate cancer metastasizes to the bone. Bisphosphonates primarily function to inhibit bone resorption and regulate bone mineralization. Therefore, when the bones are already undergoing active osteoblastic reactions, it may impact the uptake expression of ^68^Ga-DOTA-IBA PET/CT in osteoblastic metastatic lesions, thus affecting the diagnosis of bone metastasis.

We further analyzed semi-quantitative parameters of all lesions and found statistically significant differences in SUVmax and TBR between the two imaging agents. This difference is attributed to the higher uptake of ^18^F-NaF in bone and blood, possibly due to the faster clearance related to the closer resemblance of ^18^F-NaF to calcium salts compared to bisphosphonates (8). However, this higher uptake may also result in an increased risk of false positives for ^18^F-NaF. The radionuclide ^68^Ga was used to label DOTA-IBA. And, as mentioned above, the diagnostic efficacy of ^68^Ga-DOTA-IBA in prostate cancer bone metastasis has shown inconsistent similarity to that of ^18^F-NaF. This could be due to bisphosphonates’ mechanism of action, which enhances bone mineralization regulation (22, 23). Bisphosphonates exhibit non-uniform distribution in different bone tissue areas and can partially repair osteolysis lesions (24, 25). In our study, most patients had multiple bone metastases throughout the body, some of which were osteogenic. Thus, we propose that the factors affecting ^68^Ga-DOTA-IBA uptake are more likely to be related to the osteogenic reaction of the lesions, and then affect the detection of lesions. However, this view also needs to be further confirmed by larger sample size and more rigorous research.

On the other hand, The difference in SUVmax AUC between the two imaging agents was not statistically significant, as was the difference in AUC of TBR. This suggests that SUVmax and TBR may have little impact on the final diagnosis of bone metastasis in patients. Some studies have indicated that ^18^F-NaF PET/CT can detect more bone lesions than ^99m^Tc-MDP, but some scholars argue that the number of lesions may not affect clinical treatment decisions when bone metastasis is diffuse (26). Therefore, ^99m^Tc-MDP is considered sufficient for diagnosing bone metastases, supporting its diagnostic value. Similarly, although ^18^F-NaF PET/CT may detect more lesions and have a higher uptake value, the diagnostic performance of ^68^Ga-DOTA-IBA for bone metastasis is statistically consistent. Thus, when bone metastasis is diffuse, the number of lesions may not impact clinical treatment decisions.

In recent years, several radiopharmaceuticals have been utilized for the treatment of bone metastases, such as ^32^P, ^89^Sr, ^223^Ra, ^188^Re/^186^Re-HEDP, ^153^Sm-EDMTP, ^177^Lu-EDTMP, and ^177^Lu-BPAMD (27–30). While these therapeutic radiopharmaceuticals have shown promise in alleviating pain, they have not been ideal for theranostic purposes due to the lack of corresponding diagnostic analogs, with the exception of ^68^Ga/^177^Lu-BPAMD (30). The combination of ^68^Ga/^177^Lu-DOTA-IBA presents a promising set of theranostic radiopharmaceuticals and holds great potential for the management of bone metastases (13, 31, 32).

**Figure 3:**
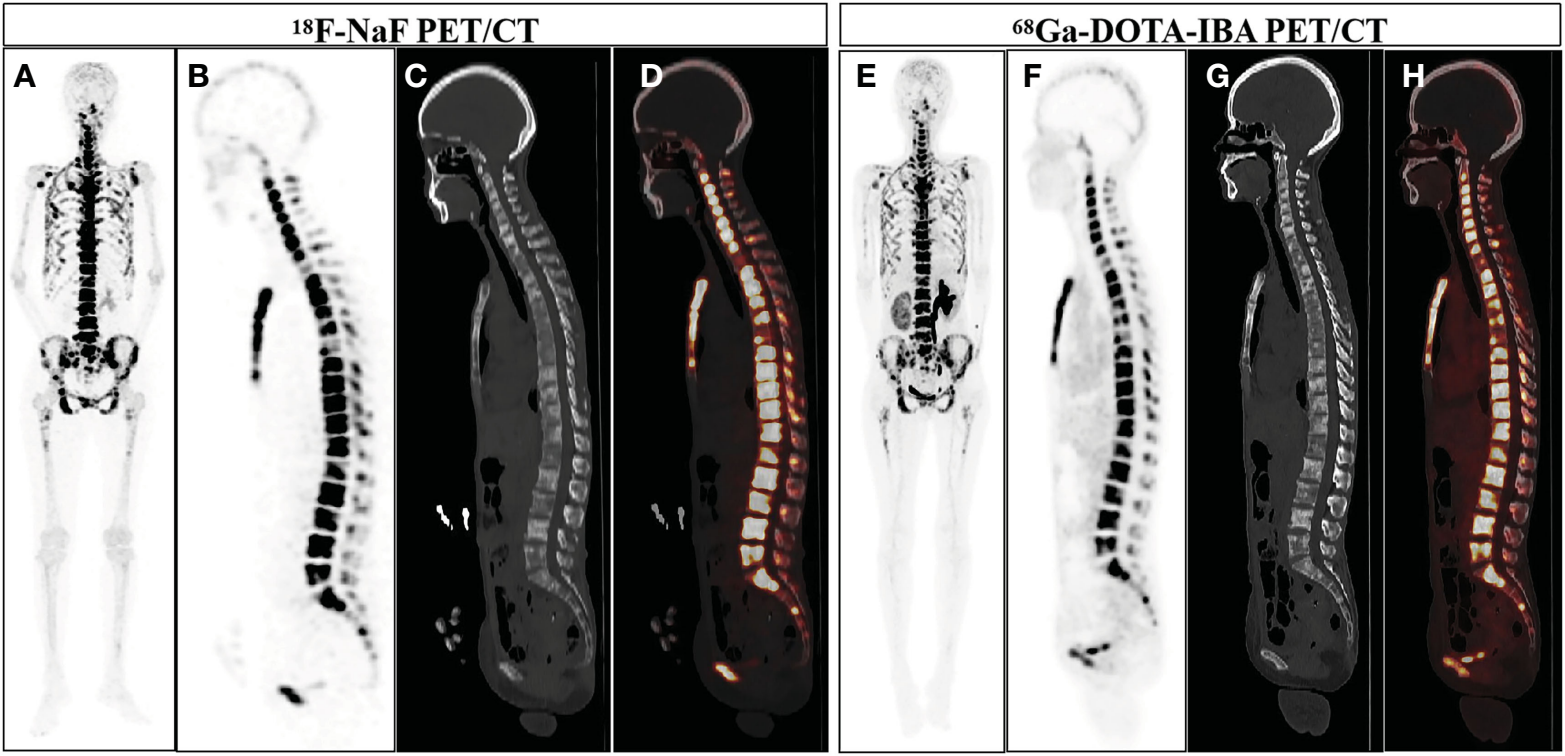
A 52-year-old woman who underwent gastric cancer surgery underwent ^18^F-NaF PET/CT examination for follow-up. The MIP image of ^18^F-NaF PET/CT **(A)** showed increased uptake of imaging agents in multiple skeletal areas throughout the body, and the sagittal views (**B**: PET image; **C**: CT scan; **D**: PET/CT fused image) more intuitively displayed bone destruction and increased uptake of imaging agents in multiple skeletal areas (SUVmax 76.5). ^68^Ga-DOTA-IBA PET/CT MIP **(E)** and the sagittal views (**F**: PET image; **G**: CT scan; **H**: PET/CT fused image) also showed increased uptake of bone imaging agents in multiple locations throughout the body (SUVmax 21.4).

Although to some extent, ^18^F-NaF PET/CT is superior to ^68^Ga-DOTA-IBA. However, in the absence of fluorine-18, ^68^Ga-DOTA-IBA PET/CT offers an option for PET radiopharmaceuticals produced by generators. More importantly, ^68^Ga-DOTA-IBA PET/CT imaging can be used for dynamic diagnosis, staging and response evaluation of bone metastasis, making the corresponding radionuclide therapy (^177^Lu-DOTA-IBA) treatment personalized. In the case illustrated in **Figure 3**, a 52-year-old woman undergoing cancer surgery received ^18^F-NaF PET/CT for follow-up. The Maximum Intensity Projection (MIP) image (A) and sagittal view (B: PET image; C: CT scan; D: fusion image) of ^18^F-NaF PET/CT revealed bone destruction and increased uptake of the imaging agent in multiple skeletal areas (SUVmax 76.5). Additionally, abnormal uptake of the imaging agent throughout the body bones was observed on the ^68^Ga-DOTA-IBA PET/CT MIP. Although the SUVmax value on the tomographic image was lower than that of ^18^F-NaF (SUVmax 21.4), this discrepancy does not impact the diagnosis of bone metastasis. For patients undergoing corresponding radionuclide treatment, this information can help in evaluating the treatment’s effectiveness.

This study has certain limitations. First, this is a retrospective analysis of a relatively small patient group (n=24), so these results were susceptible to selection bias. Secondly, due to technical and ethical issues, biopsy was not performed on all bone metastases regarding the biopsy of bone lesions in individuals with multiple suspected metastases, which limits the reference standards. Finally, the diversity of primary diagnosis of patients in our patient group may interrupt the homogeneity, the number of cases of different primary solid tumors in our study was relatively small, so we only made a preliminary analysis of the subgroup, and because of the complex mechanism of bone metastasis, we did not conduct a subgroup analysis of osteolytic and osteogenic metastasis.

## Conclusion

Overall, while ^68^Ga-DOTA-IBA may not be as effective as ^18^F-NaF as a professional diagnostic bone imaging agent, statistically speaking, the final diagnosis results for all lesions are consistent. Its labeled nuclide is obtained from the generator, and the precursor retains the advantage of high bone adsorption rate of IBA, enabling the ^68^Ga/^177^Lu-DOTA-IBA theranostics pair to offer potential clinical benefits. These findings are preliminary, and further large-scale prospective trials are necessary to fully assess the value of ^68^Ga-DOTA-IBA in detecting bone metastases or different bone metastatic lesions in patients with various types of cancer.

## Data availability statement

The original contributions presented in the study are included in the article/supplementary material. Further inquiries can be directed to the corresponding authors.

## Ethics statement

The studies involving humans were approved by the Ethics Committee of the Affiliated Hospital of Southwest Medical University (Ethics committee approval No.: KY2022114; Clinical trial registration No.: ChiCTR2200064487). The studies were conducted in accordance with the local legislation and institutional requirements. Written informed consent for participation in this study was provided by the participants’ legal guardians/next of kin. Written informed consent was obtained from the individual(s) for the publication of any potentially identifiable images or data included in this article.

## Author contributions

JD: Conceptualization, Data curation, Formal analysis, Methodology, Writing – original draft. JY: Methodology, Resources, Writing – original draft. YW: Data curation, Resources, Writing – review & editing. GL: Methodology, Writing – review & editing. YC: Conceptualization, Writing – review & editing.
